# Author Correction: Ultrafast switch-on dynamics of frequency-tuneable semiconductor lasers

**DOI:** 10.1038/s41467-018-07629-5

**Published:** 2018-11-30

**Authors:** Iman Kundu, Feihu Wang, Xiaoqiong Qi, Hanond Nong, Paul Dean, Joshua R. Freeman, Alexander Valavanis, Gary Agnew, Andrew T. Grier, Thomas Taimre, Lianhe Li, Dragan Indjin, Juliette Mangeney, Jérôme Tignon, Sukhdeep S. Dhillon, Aleksandar D. Rakić, John E. Cunningham, Edmund H. Linfield, A. Giles Davies

**Affiliations:** 1 School of Electronic and Electrical Engineering, University of Leeds, Leeds, LS2 9JT UK; 2 Laboratoire Pierre Aigrain, Département de physique de l’ENS, École normale supérieure, PSL Research University, Université Paris Diderot, Sorbonne Paris Cité, Sorbonne Universités, UPMC Univ. Paris 06, CNRS, 75005 Paris, France; 3 School of Information Technology and Electrical Engineering, The University of Queensland, Brisbane, QLD 4072 Australia; 4 School of Mathematics and Physics, The University of Queensland, Brisbane, QLD 4072 Australia

Correction to: *Nature Communications*; 10.1038/s41467-018-05601-x; published online 06 August 2018

The original version of this Article contained an error in the Acknowledgements, which incorrectly omitted the following: ‘We also acknowledge support from the Australian Research Council’s Discovery Projects Funding Scheme (Grant DP 160 103910).’ This has been corrected in both the PDF and HTML versions of the Article.

